# Directional Polarization of a Ferroelectric Intermediate Layer Inspires a Built‐In Field in Si Anodes to Regulate Li^+^ Transport Behaviors in Particles and Electrolyte

**DOI:** 10.1002/advs.202402915

**Published:** 2024-04-19

**Authors:** Ming Liu, Wenqiang Xu, Shigang Liu, Bowen Liu, Yang Gao, Bin Wang

**Affiliations:** ^1^ CAS Key Laboratory of Nanosystem and Hierarchical Fabrication National Center for Nanoscience and Technology Beijing 100190 P. R. China; ^2^ University of Chinese Academy of Sciences Beijing 100039 P. R. China; ^3^ State Key Laboratory for Advanced Metals and Materials School of Materials Science and Engineering University of Science and Technology Beijing Beijing 100083 P. R. China; ^4^ Key Laboratory of Bio‐based Material Science and Technology of Ministry of Education Engineering Research Center of Advanced Wooden Materials of Ministry of Education College of Material Science and Engineering Northeast Forestry University Harbin 150040 P. R. China

**Keywords:** BaTiO_3_, electric field polarization, ferroelectric materials, lithium‐ion batteries, silicon anode

## Abstract

The silicon (Si) anode is prone to forming a high electric field gradient and concentration gradient on the electrode surface under high‐rate conditions, which may destroy the surface structure and decrease cycling stability. In this study, a ferroelectric (BaTiO_3_) interlayer and field polarization treatment are introduced to set up a built‐in field, which optimizes the transport mechanisms of Li^+^ in solid and liquid phases and thus enhances the rate performance and cycling stability of Si anodes. Also, a fast discharging and slow charging phenomenon is observed in a half‐cell with a high reversible capacity of 1500.8 mAh g^−1^ when controlling the polarization direction of the interlayer, which means a fast charging and slow discharging property in a full battery and thus is valuable for potential applications in commercial batteries. Simulation results demonstrated that the built‐in field plays a key role in regulating the Li^+^ concentration distribution in the electrolyte and the Li^+^ diffusion behavior inside particles, leading to more uniform Li^+^ diffusion from local high‐concentration sites to surrounding regions. The assembled lithium‐ion battery with a BaTiO_3_ interlayer exhibited superior electrochemical performance and long‐term cycling life (915.6 mAh g^−1^ after 300 cycles at a high current density of 4.2 A g^−1^). The significance of this research lies in exploring a new approach to improve the performance of lithium‐ion batteries and providing new ideas and pathways for addressing the challenges faced by Si‐based anodes.

## Graphical abstract

A ferroelectric (BaTiO_3_) interlayer and field polarization treatment are introduced to set up a built‐in field, which optimizes the transport mechanisms of Li^+^ in solid and liquid phases and thus enhances the rate performance and cycling stability of Si anodes. The significance of this research lies in providing new ideas for addressing the challenges faced by Si‐based anodes.

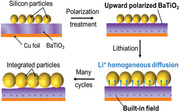

## 1 Introduction

For lithium‐ion batteries (LIBs), ion transport primarily involves the transport in electrodes, electrolytes, and interfaces. The transport of solvated ions in electrolytes is driven by an electric field gradient and follows Ohm's law.^[^ ^1^ ^]^ Besides the electric field gradient, concentration gradient and electrochemical potential gradient are also driving forces of Li^+^ transport in the solid phase. Generally, the ion transport resistance in the surface and interior of electrode materials is much greater than that in electrolytes.^[^ ^2^ ^]^ Therefore, the Li^+^ diffusion process in the solid phase is often the rate‐determining step during charge/discharge processes.^[^ ^3^, ^4^ ^]^ At high‐rate processes, a high electric field and concentration gradient form on the surface of active materials, which is adverse to the structural and cycling stability of electrodes.^[^ ^5^, ^6^, ^7^, ^8^ ^]^ Recently, several methods such as altering the structural features of electrode materials and introducing an internal built‐in field have been proposed to optimize the Li^+^ transport behaviors in the solid phase.^[^ ^9^, ^10^, ^11^ ^]^ However, how to optimize the nonlinear spatial distribution of concentration and electric field gradients still faces major challenges.^[^ ^12^, ^13^ ^]^

Ferroelectric materials are known for a high dielectric constant and a polarizable attribute and have the potential to generate built‐in fields.^[^ ^14^, ^15^, ^16^ ^]^ By designing appropriate structures, it is possible to effectively regulate ion migration between electrodes and electrolytes, which is emerging as a new research direction.^[^ ^17^, ^18^, ^19^ ^]^ Recently, a BaTiO_3_ layer has been introduced into electrochemical energy storage systems to regulate ion transport dynamics. For example, Zou et al. achieved self‐accelerated ion migration at the interface of BaTiO_3_/Zn by incorporating a thin film coating of a ferroelectric polymer‐inorganic composite into zinc metal anode batteries.^[^ ^20^ ^]^ Guo et al. achieved uniform hexagonal and cubic lithium deposition at high currents by introducing a BaTiO_3_‐based ferroelectric substrate into lithium metal batteries.^[^ ^21^ ^]^ Research concerning the impact of nanoparticle additives or nanofilm coatings on the charge/discharge capacity of both anodes and cathodes in LIBs primarily emphasizes the role of additives in enhancing capacity and improving cycle life.^[^ ^22^, ^23^, ^24^ ^]^ However, the mechanism of ion transport in electrodes and electrolytes under a ferroelectric built‐in field is still indefinable at the microscale, particularly regarding how the built‐in field affects Li^+^ concentration distribution and solid/liquid‐state Li^+^ diffusion dynamics.^[^ ^25^, ^26^ ^]^

In this study, we used a dual‐coating process to prepare a ferroelectric interlayer (BaTiO_3_/C) and an active layer (Si/C) on copper foil successively to obtain a high‐performance Si anode. The BaTiO_3_ interlayer could mitigate the pulverization of Si particles as caused by the volume expansion during lithiation. More importantly, it was found that when an upward polarization treatment was applied to the BaTiO_3_ interlayer, a high reversible capacity of 1500.8 mAh g^−1^ could be achieved under fast discharging and slow charge conditions in a half‐cell, which is valuable for commercial battery uses such as in electric cars. Finite element modeling (FEM) was conducted to study performance enhancement derived from the BaTiO_3_ interlayer. The upward polarized BaTiO_3_ provides a new driving force for Li^+^ migration in Si particles, resulting in more uniform diffusion from local high‐concentration sites to surrounding areas. Therefore, the Si/C anode with a BaTiO_3_ interlayer exhibits superior lithium storage performance and long cycling life. This study provides new ideas and approaches for regulating Li^+^ transport dynamics in Si‐based anodes and exploring the application of ferroelectric materials in LIBs.

## 2 Results and Discussion

**Figure 1:**
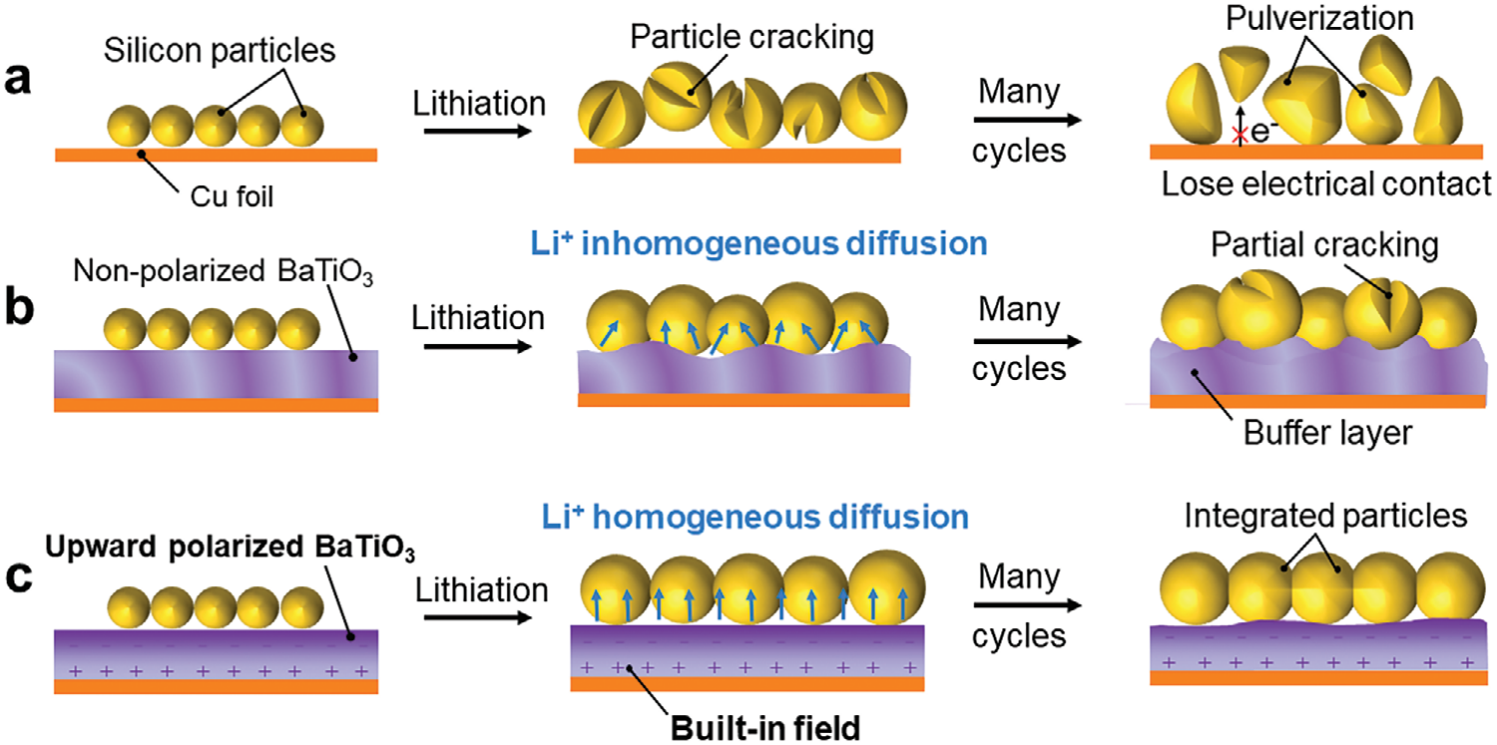
Schematic diagram of the changes in lithiation processes of different Si anodes. a) Si particles, b) Si particles with a non‐polarized BaTiO_3_ interlayer, and c) Si particles with an upward polarized BaTiO_3_ interlayer.

During lithiation, a volumetric expansion of up to 300–400% occurs in Si, which can easily cause particle fracture and significant capacity loss due to particle pulverization and loss of electrical contact with the current collector after multiple cycles (**Figure** **1**a).^[^ ^27^, ^28^, ^29^, ^30^ ^]^ A BaTiO_3_ interlayer introduced between the Si anode and copper current collector could serve as a lateral buffer. Before corona poling, the dipoles in BaTiO_3_ are randomly arranged, resulting in the absence of a uniform built‐in field. However, after high‐voltage corona poling treatment, the ferroelectric nature of BaTiO_3_ causes the dipoles in BaTiO_3_ to align along the applied electric field direction, thus achieving the separation of positive and negative charges (Figure S1, Supporting Information). To induce upward polarization in BaTiO_3_, an upward electric field was applied to the copper foil coated with BaTiO_3_. This process facilitated the uniform establishment of an electric field within the BaTiO_3_ layer, thus promoting the uniform accumulation of Li^+^ on the surface of the BaTiO_3_ layer. Repulsion of Li^+^ at positively charged sites and attraction of Li^+^ at negatively charged sites coexist at the Si/BaTiO_3_ interfaces, leading to the inhomogeneous diffusion of Li^+^ and the partial fracture of Si particles (Figure 1b). Therefore, upward polarization treatment was used to align the electric dipoles and generate a negatively charged surface on BaTiO_3_. In this way, Li^+^ is driven to homogeneously diffuse across the interfaces and Si particles could possibly maintain integrity after cycling (Figure 1c).^[^ ^31^ ^]^

Based on the above principles, a Si‐based composite electrode was constructed using a simple dual‐layer coating process, which consists of a silicon‐carbon (Si/C) active layer and a BaTiO_3_ interlayer (Figure S1, Supporting Information). BaTiO_3_ particles used in this work have an average diameter of 100–250 nm with regular shapes and are composed of a tetragonal phase with a ferroelectric feature (Figures S2,S3, Supporting Information). The XRD patterns of the double‐layer electrodes before and after 100 cycles at 2.1 A g^−1^ revealed that the XRD peaks of Si disappeared after cycling, indicating the transformation of Si from a crystalline to an amorphous state. The peak of BaTiO_3_ shifted to the right by ≈0.3°, indicating its mechanical compression by the electrochemical reaction of Si, resulting in a decrease in lattice constants (Figure S3, Supporting Information). These results indicate the contribution of the mechanical deformation of BaTiO_3_ and the resulting piezoelectricity to the battery performance. Besides, the polarization mode of BaTiO_3_ is also an important factor for its application. Therefore, three kinds of polarization modes were considered in this work, namely non‐polarization, upward polarization (built‐in field points towards BaTiO_3_), and downward polarization (built‐in field points towards Cu foil).

**Figure 2:**
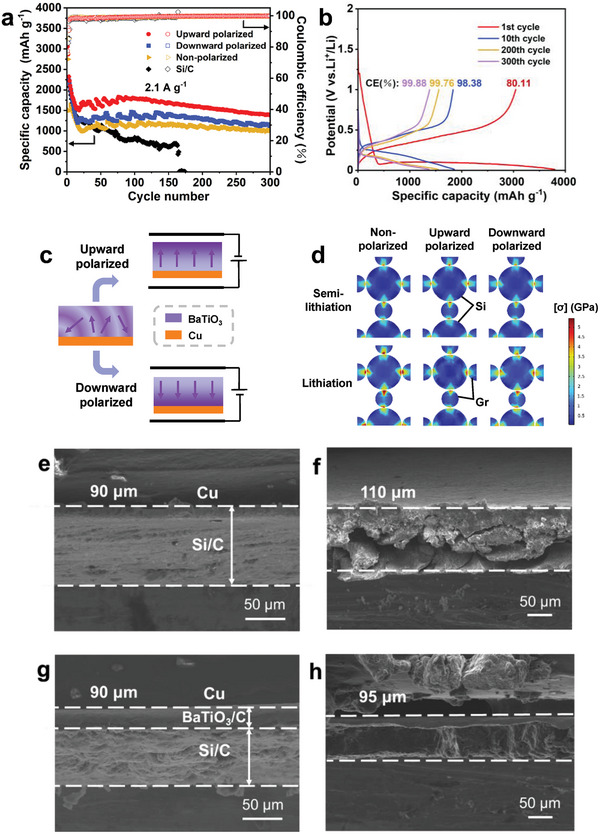
Cycling performance, mechanical simulation, and morphological characterization of Si‐based anodes. a) Cycling performance of Si/C, non‐polarized, upward polarized, and downward polarized electrodes at 2.1 A g^−1^. b) Charge/discharge profiles of the upward polarized electrode. c) Schematic diagram of dipole arrangement under upward and downward polarization. d) von Mises stress of non‐polarized, upward polarized, and downward polarized samples at different lithiation states. e,f) SEM images of the Si/C electrode before (e) and after (f) 50 cycles. g,h) SEM images of the upward polarized electrode before (g) and after (h) 50 cycles.

Constant current cycling tests using CR2032 coin‐type half‐cells were conducted to investigate the effect of electric field polarization treatment on the electrochemical performance of Si/C anodes with a BaTiO_3_ interlayer. As shown in **Figure** **2**a, the single‐layer Si/C anode shows rapid capacity decay and breaks down after 166 cycles at 0.5 C (1 C = 4200 mA g^−1^). In contrast, Si/C anodes with a BaTiO_3_ interlayer show superior cycling durability. Specifically, the upward polarized sample shows an initial specific capacity of 3041.9 mAh g^−1^ and an initial Coulombic efficiency of 80.11%, which is higher than that of the typical Si/C electrode (Figure 2b). Then, its specific capacity decreases to 1834.3, 1550.3, and 1389.5 mAh g^−1^ after 10, 200, and 300 cycles, respectively, all of which are larger than those of the downward polarized and non‐polarized samples. It can be seen that rapid capacity decay primarily occurs in initial cycles, while the subsequent cycles show relatively stable performance, with only a 10% capacity decrease from the 200th to 300th cycle. These findings are attributed to the uniform alignment of dipoles in the BaTiO_3_ interlayer, especially under upward polarization (Figure 2c). This induces a low surface potential and thus enhances the homogeneous diffusion of Li^+^ and the structural stability of Si/C anodes during cycling. Furthermore, we conducted Kelvin probe force microscopy (KPFM) to evaluate the surface potential of different polarized samples. As shown in Figure S4a–c (Supporting Information), the KPFM test results indicated that the selective polarization procedures have a noticeable influence on the surface potentials of different samples. The measured surface potentials of the downward polarized, non‐polarized, and upward polarized samples were ≈750, ≈0, and ≈−750 mV, respectively (Figure S4d, Supporting Information). This provides direct evidence for the attraction of Li^+^ by a low surface potential of the upward polarized sample. Besides, a full cell consisting of an LFP (lithium iron phosphate) cathode and the upward polarized anode was assembled (Figure S5, Supporting Information).^[^ ^32^ ^]^ It maintains 93.1 mAh g^−1^ after 50 cycles, suggesting the feasibility of the dual‐layer polarization treatment.

To further investigate the mechanism of improved electrochemical performance derived from the BaTiO_3_ interlayer, FEM simulation studies were conducted using Comsol Multiphysics software. As shown in Figure S6 (Supporting Information), the Si/C composite electrode was located at the top left, and the LFP cathode was located at the bottom right. At the anode side, a BaTiO_3_/C interlayer was placed near Cu foil to get close to the actual electrode structure.^[^ ^33^ ^]^ Only the discharge process was simulated, with a constant current of 1 mA m^−2^ for rapid discharge lasting 10 min. The simulated voltage curve matched well with the experimental one, indicating good consistency between simulations and experiments (Figure S7, Supporting Information).^[^ ^34^ ^]^

Then, the local stress distribution around Si and graphite (Gr) particles was analyzed. As shown in Figure 2d, the Si and Gr particles were arranged alternately, with large circles representing Si and small circles representing Gr. As lithiation proceeds, the Si and graphite (Gr) particles undergo volume expansion. To describe this process, we coupled the diffusion and mechanical model by using concentration and strain as two parameters, and thus volume expansion could be expressed as a function of local solid lithium concentration by an interpolation method.^[^ ^35^, ^36^, ^37^ ^]^ Subsequently, the von Mises stress of Si and Gr particles at different lithiation degrees was calculated. It can be seen that the maximum value of von Mises stress occurs at the contact points, while the stress at free boundaries and inside particles is relatively small. At the semi‐lithiation state, the stress values under three polarization states are similar. However, as lithiation proceeds, the stress of the non‐polarized sample significantly increases, especially at the contact points where the von Mises stress exceeds 5.0 GPa, which is particularly evident in Gr particles. Conversely, the stress under upward and downward polarization states is relatively small and has no significant change during lithiation. These results indicate that the polarized BaTiO_3_ can regulate Li^+^ diffusion to alleviate excessive local stress in anode particles, thereby reducing the fracture and pulverization of active particles and improving the long‐term cyclic stability of anodes.^[^ ^38^, ^39^ ^]^

To evaluate the morphology change of Si/C anodes, their cross‐sectional images were obtained using scanning electron microscopy (SEM). As shown in Figures 2e–h, the thickness of the single‐layer Si/C anode increased by around 22% after 50 cycles at 2 A g^−1^, and large cracks appeared. In contrast, the upward polarized electrode with a BaTiO_3_ interlayer remains intact after cycling, with no significant cracks. To ascertain the long‐term cycle stability of bilayer polarized electrodes, we acquired SEM images of electrode cross‐sections before and after different cycles for both non‐polarized and upward polarized conditions. As depicted in Figure S8 (Supporting Information), the cross‐sectional thickness of the upward polarized electrode increased from 90 µm to 130 µm after 300 cycles, yet devoid of significant internal fissures, indicating preserved material integrity. The non‐polarized electrode retained its integrity after 100 cycles but exhibited minor cracking following 300 cycles. This result indicates that the BaTiO_3_ interlayer could act as a lateral buffer layer to release the stress generated in the Si/C layer, and thus keep the particle integrity and enhance cycling performance.

**Figure 3:**
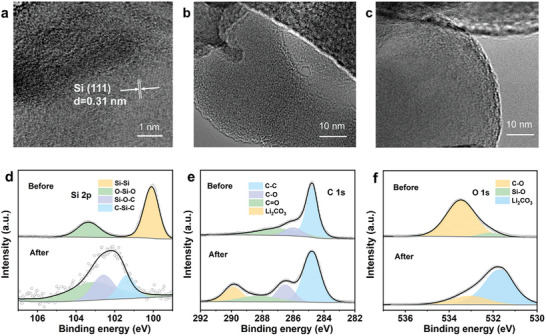
Interfacial and componential characterization of different Si anodes. a,b) HRTEM of nano‐silicon particles of Si/C before and after 100 cycles. c) HRTEM of nano‐silicon particles of upward polarized samples after 100 cycles. d) Si 2p XPS spectra of upward polarized samples before and after 100 cycles. e) C 1s XPS spectra of upward polarized samples before and after 100 cycles. f) O 1s XPS spectra of upward polarized samples before and after 100 cycles.

To further investigate the evolution of Si/C anodes during cycling, a non in situ characterization of nano‐silicon particles was conducted utilizing transmission electron microscopy (TEM). The high‐resolution (HRTEM) image of the original Si/C revealed a lattice spacing of 0.31 nm corresponding to the (111) plane of Si (**Figure** **3**a).^[^ ^40^ ^]^ After 100 cycles, the nano‐silicon particles undergo a transition from a crystalline to an amorphous state, accompanied by observed silicon particle boundary fusion in the Si/C anodes (Figure 3b). Conversely, the double‐layer upward polarized samples demonstrated improved preservation of the silicon particle morphology (Figure 3c). X‐ray photoelectron spectroscopy (XPS) was employed for elemental analysis and valence state determination of upward polarized electrodes. The original upward polarized electrode exhibits silicon bonding as Si−Si (99.9 eV) and O−Si−O (103.6 eV), indicative of a SiO_2_ layer on silicon particle surfaces (Figure 3d).^[^ ^41^ ^]^ In contrast, the Si 2p spectrum of the upward polarized sample after cycling reveals the emergence of three valence states: Si^2+^ (101.5 eV), Si^3+^ (102.5 eV), and Si^4+^ (103.6 eV), suggesting the formation of Si−O−C and C−Si−C bonds. The C 1s spectrum of the original electrode shows distinct peaks at 284.4, 286.6, and 288 eV, corresponding to C−C, C−O, and C═O, respectively (Figure 3e).^[^ ^42^ ^]^ Notably, a significant peak appears at 289.7 eV in the C 1s spectrum of the cycled upward polarized sample due to the presence of carbonate compounds (labeled as Li_2_CO_3_). This aligns well with the O 1s spectrum, reflecting the reduction and consumption of electrolytes (ethylene carbonate (EC) and diethyl carbonate (DEC) in this work) at the electrode‐electrolyte interface (Figure 3f), where Li_2_CO_3_ emerges as the principal component of the solid‐electrolyte interphase (SEI).^[^ ^43^ ^]^ The presence of inorganic compounds (Li_2_O, Li_2_CO_3_) serves to inhibit further decomposition of the electrolyte and contributes to the formation of a stable SEI layer. This phenomenon is advantageous in enhancing the interfacial stability of electrode materials, thereby improving the long‐term cyclic performance of batteries.

**Figure 4:**
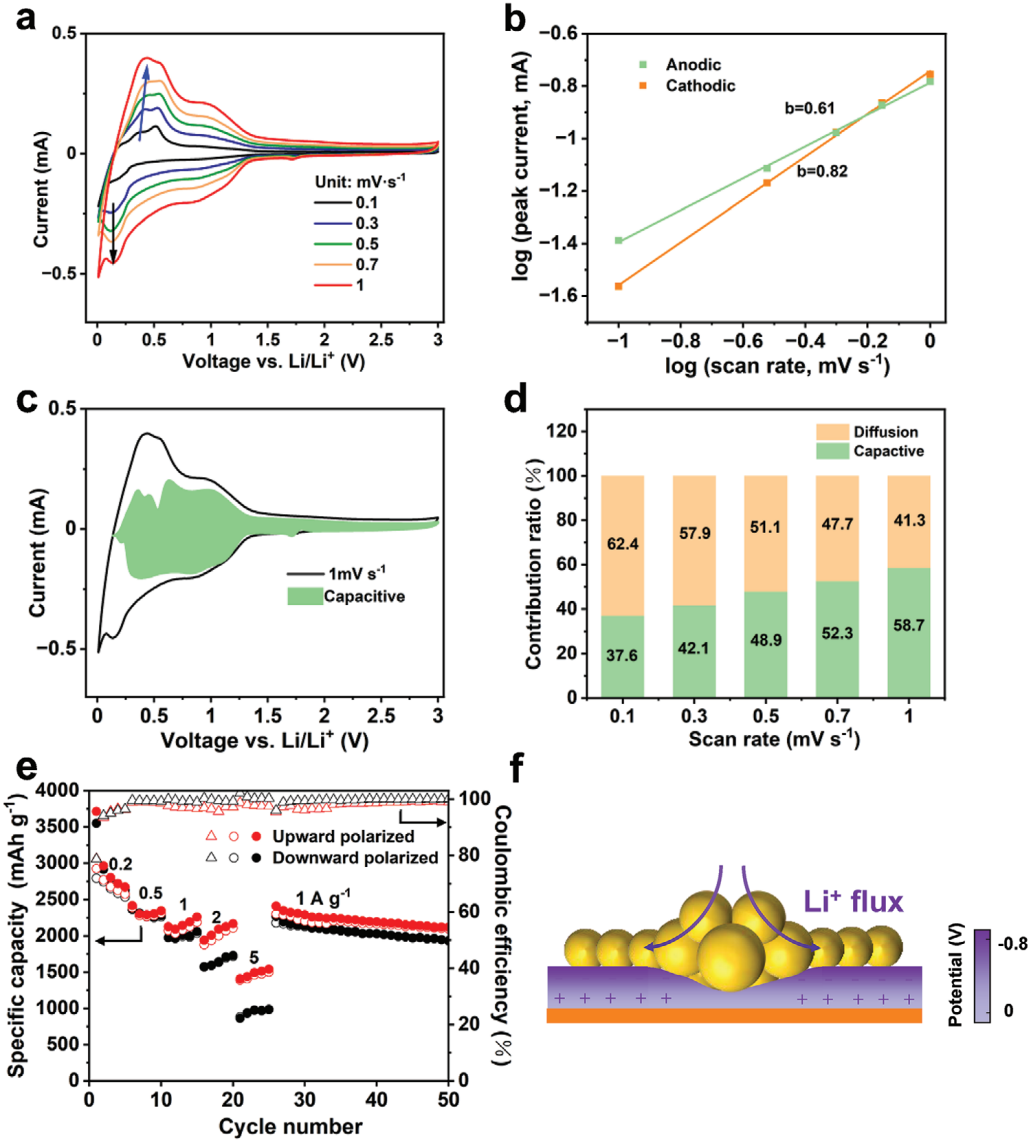
Kinetics characterization and rate capability. a) CV profiles of the upward polarized sample at different scan rates. b) Correlations between the logarithm of peak current and the logarithm of scan rate. c) CV curve and capacitive proportion to the charge storage of the upward polarized sample at 1.0 mV s^−1^. d) Normalized proportion ratio of capacitive and diffusion‐controlled capacities at various scan rates. e) Rate performance of upward polarized and downward polarized samples at different discharge rates and a constant charge rate of 0.2 A g^−1^. f) Schematic diagram of the piezoelectric effect at the BaTiO_3_ layer of the upward polarized sample.

In order to demonstrate the influence of the polarization mode of BaTiO_3_ to Li^+^ transport, cyclic voltammetry (CV) curves of the upward and downward polarized samples were tested at different scan rates.^[^ ^12^, ^44^ ^]^ As shown in **Figure** **4**a, these curves exhibit similar shapes and slight peak shifts, indicating that the upward polarized sample has fast reaction kinetics. Figure 4b illustrates the linear relationships between the logarithmic values of peak current and scan rate for both cathodic and anodic peaks. The electrochemical reaction kinetics were then analyzed using a power‐law relation: *i*  =  *av^b^*. In this formula, a value of b = 0.5 indicates that the electrochemical behavior is controlled by semi‐infinite linear diffusion, while a value of b = 1 indicates that the charge storage process is controlled by capacitive behaviors.^[^ ^45^, ^46^ ^]^ The linear fitting results indicate that the upward polarized sample has a b value of 0.82 and 0.61 for cathodic and anodic peaks, respectively. The findings indicate that the process of lithiation is predominantly characterized by capacitive behaviors. It is noteworthy that the b value for lithiation is higher than that for delithiation, suggesting that the upward polarized sample exhibits faster discharge and slower charge performance. Furthermore, the proportion of capacitive lithium storage behaviors can be quantitatively evaluated using the formula *i*  = *k*_1_ *v* + *k*_2_ *v* ^1/2^.^[^ ^47^ ^]^ This formula indicates that the electrochemical reaction current consists of two parts: one is the capacitive effect (*k*_1_ *v*), and the other is the diffusion‐controlled reaction (*k*_2_ *v* ^1/2^). By calculating the values of *k*_1_ and *k*_2_, the current proportions originating from capacitive behaviors and Li^+^ diffusion‐controlled processes at specific potentials can be determined. As shown in Figure 4c, the shaded area represents the proportion of the capacitive current in the overall current response. The upward polarized sample demonstrates a dominant capacitive contribution (58.7%) at 1.0 mV s^−1^. The ratio of the capacitive part increases with the scan rate, indicating its excellent rate performance for lithium storage (Figure 4d). The capacitive contribution of the downward polarized sample is less than that of the upward polarized sample at all scan rates (Figure S9, Supporting Information). Therefore, the polarization direction of the BaTiO_3_ interlayer plays a crucial role in regulating Li^+^ transport and facilitating reaction kinetics.

Besides, the rate performance of upward‐ and downward‐polarized electrodes was tested at a constant charging rate but different discharging rates. As shown in Figure 4e, the upward polarized electrode shows significantly higher capacity retention at high discharge rates, since upward polarization provides abundant Li^+^ adsorption sites to enhance the rapid discharge capability. When the discharge current increases to 5 A g^−1^, the upward polarized electrode delivers a specific capacity of 1500.8 mAh g^−1^, which is higher than that of the downward polarized electrode (985.3 mAh g^−1^). It is worth noting that the downward polarized electrode exhibits higher Coulombic efficiency than that of the upward polarized electrode at 2 A g^−1^ and 5 A g^−1^. This can be attributed to the repulsion of Li^+^ by the downward polarized BaTiO_3_, thus promoting the diffusion and migration of Li^+^. On the contrary, the attraction of Li^+^ by the upward polarized BaTiO_3_ hinders the migration of Li^+^ from the anode to the cathode. Therefore, the polarization treatment of BaTiO_3_ can improve the lithium storage capacity of the double‐layer Si/C anode and alter the charge and discharge characteristics of battery devices.

In addition, the ferroelectric BaTiO_3_ also possesses piezoelectric properties, and the diffusion direction of Li^+^ can be regulated by changing the built‐in field during lithiation.^[^ ^16^, ^48^ ^]^ Upon polarization of the ferroelectric material, a built‐in electric field arises due to the displacement of Ti ion from the central position within the [TiO_6_] octahedra (inverse piezoelectric effect, Figure S10, Supporting Information). During lithiation, volumetric expansion of silicon particles induces compressive stress, which partially reverses the deformation of the BaTiO_3_ and diminishes the local built‐in field (positive piezoelectric effect). This modulation facilitates the dispersion of Li^+^ to alternate sites, dynamically regulating the Li^+^ transport. After upward polarization treatment within a 1 kV cm^−1^ electric field, BaTiO_3_ exhibits a surface polarization potential of −0.8 V.^[^ ^49^ ^]^ As shown in Figure 4f, when Si particles experience significant volume expansion during lithiation, the local built‐in field inside the BaTiO_3_ interlayer is altered. Subsequently, the diffusion direction of Li^+^ also changed, thereby alleviating excessive internal stress caused by volume expansion.^[^ ^50^ ^]^

**Figure 5:**
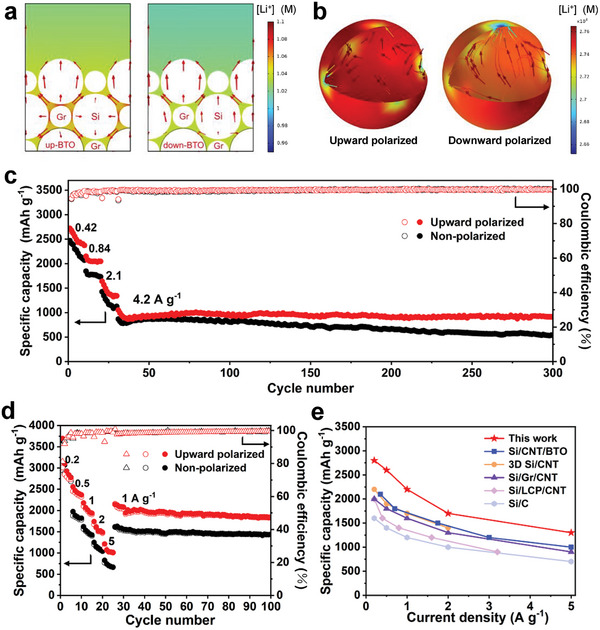
High current electrochemical simulation and performance. a) Simulation results of Li^+^ concentration distribution in the electrolyte of upward‐ and downward‐polarized electrodes. The red arrow indicates the direction of Li^+^ flux. b) Simulation results of Li^+^ concentration distribution in a Si particle of upward‐ and downward‐polarized electrodes. The red arrow in (a) and (b) indicates the direction of Li^+^ flux. c,d) Cycling performance and rate performance of upward polarized and non‐polarized electrodes. e) Rate performance comparison between the upward polarized electrode and recently reported Si‐based anodes.

In order to visually demonstrate the role of the BaTiO_3_ interlayer in Li^+^ diffusion dynamics, we extracted the X‐Y plane of the 3D geometric model and observed the Li^+^ concentration and flux (red arrows) in the electrolyte under different polarization states. According to FEM simulations, the upper surface potential of the upward‐ and downward‐polarized BaTiO_3_ layers is −0.8V and 0.8V, respectively, which is consistent with the KPFM results (Figures S4 and S11, Supporting Information). Then, the polarization potential was used as the boundary potential at the interface between the BaTiO_3_ layer and the Si/C layer. Up‐BTO and down‐BTO particles were named BaTiO_3_ particles in the upward and downward polarization states, respectively. We simulated the discharge process with a constant current of 1 mA m^−2^ for rapid discharge for 10 mins. As shown in **Figure** **5**a, the upward polarization state presents a higher Li^+^ concentration in the electrolyte between Si and Gr particles than that of the downward polarization state, thus facilitating rapid discharge and battery cycling stability. Additionally, a Si particle adjacent to the BaTiO_3_ layer was selected. Figure 5b shows the Li^+^ concentration distribution on the particle surface and the Li^+^ flux inside the particle after being sliced. It can be seen that the overall Li^+^ concentration of the Si particle near the up‐BTO is higher than that around the down‐BTO, indicating a faster lithium insertion process.

To further demonstrate the advantages of upward polarization treatment, high‐rate cycling tests on half‐cells were conducted. As shown in Figure 5c, the upward polarized electrode remains a reversible capacity of 915.6 mAh g^−1^ after 300 cycles at 1C, along with a capacity retention of 87.1%. In contrast, the non‐polarized shows a capacity retention of only 61.4%, therefore revealing the highly stable cycling performance of the upward polarized electrode. Furthermore, the upward polarized electrode exhibits significantly higher specific capacities at 0.5, 1, 2, and 5 A g^−1^ than the non‐polarized samples (Figure 5d). When the current density returns to 1 A g^−1^, the upward polarized electrode delivers a specific capacity of 1828.2 mAh g^−1^ after 100 cycles, which is 400 mAh g^−1^ higher than that of the non‐polarized electrode. Even compared with the well‐designed Si‐based anodes recently reported, the upward polarized electrode still presents high electrochemical performance (Figure 5e).^[^ ^16^, ^29^, ^30^, ^38^, ^39^ ^]^ The above experimental and simulation results indicate that the built‐in field in the upward polarized BaTiO_3_ layer can regulate Li^+^ distribution in the electrolyte and inside Si particles, thus mitigating the high electric field and concentration gradients on the surface of Si particles. As a result, the long‐term electrochemical performance of Si/C anodes was greatly improved, especially at high rates.

## 3 Conclusion

In summary, we used a simple double‐layer coating method to build a standard model combining a lithium‐storage Si/C anode and a ferroelectric BaTiO_3_ interlayer, which enables the study of the directional built‐in field effects on lithium‐ion transport behaviors. In detail, the polarization mode of the BaTiO_3_ layer was altered by applying external polarization treatment, which generated upward‐ and downward‐polarized built‐in fields and regulated the distribution and diffusion of Li^+^ in the electrolyte and electrode. FEM simulations indicated that the upward polarized BaTiO_3_ drives Li^+^ to migrate in Si particles, thus leading to more uniform Li^+^ diffusion from local high‐concentration sites to surrounding regions. Furthermore, the BaTiO_3_ layer could buffer the volume expansion of Si during lithiation due to the alteration of the built‐in field caused by its compressive deformation. As a result, the upward polarized Si/C anode achieved a high reversible capacity of 1500.8 mAh g^−1^ under fast discharging and slow charge conditions in a half‐cell and showed stable high‐rate cycling performance (915.6 mAh g^−1^ after 300 cycles at 2.1 A g^−1^). This work explains the working mechanism of ferroelectric materials when being used in lithium‐ion batteries and shows more opportunities for interdisciplinary research.

## 4 Experimental Section

### Fabrication of Polarized Double‐Layer Electrode

Commercial tetragonal BaTiO_3_ powder was mixed with Super P nanoparticles at the ratio of 80:20 and fully ground for 60 mins. The BaTiO_3_/C mixture and polyvinylidene fluoride (PVDF) binder were magnetically mixed in N‐methyl‐2‐pyrrolidone (NMP) at a mass ratio of 80:20 to form a slurry. The obtained slurry was evenly coated on a piece of Cu foil (25 µm thickness) and then dried in a vacuum oven at 80 °C for 12 h to remove solvent. High voltage was applied to the Cu foil coated with BaTiO_3_ by the corona polarization method for directional polarization treatment. As illustrated in Figure S1 (Supporting Information), the voltage was set to 15 kV, the distance from the stainless steel tip to the Cu foil was set to 15 cm, and the polarization time was 12 h. The sample was defined as downward polarization when the Cu foil side was on top, and upward polarization when the BaTiO_3_ side was on top. Subsequently, nano‐silicon (50–80 nm), Super P, and lithium polyacrylate (LiPAA) binder were adequately mixed and ground in NMP at the ratio of 63:27:10 to form a slurry. The obtained slurry was evenly coated above the polarized BaTiO_3_ layer and then dried in a vacuum oven at 80 °C for 12 h to remove solvent. Unless otherwise noted, the typical mass loading of Si was 0.7–0.8 mg cm^−2^.

### Material Characterization

The morphological and structural characterizations of all samples were carried out by field emission scanning electron microscopy (FE‐SEM, Hitachi SU8220) and field emission transmission electron microscopy (FE‐TEM, FEI Tecnai G2 F20 U‐TWIN). The phase of BaTiO_3_ was measured by XRD (D/MAX‐TTRIII, CBO) between 5° and 80° with Cu Kα radiation (λ = 0.15418 nm). XPS measurements were performed on an ESCALAB250Xi apparatus with an Al Kα X‐ray source. The surface potential of different polarized samples was measured by Kelvin probe force microscopy (KPFM, Bruker Dimension Icon).

### Electrochemical Characterization

All the electrodes were degassed in a vacuum at 60 °C for at least 2 h before use. CR2302 coin‐type half‐cells were assembled in an Ar‐filled glove box (<0.1 ppm of oxygen and water) with lithium foil as the counter electrode. The electrolyte was 1 M LiPF_6_ in 1:1 (v/v) ethylene carbonate (EC) and diethyl carbonate (DEC) with 10% fluoroethylene carbonate (FEC), and the separator was porous polypropylene membrane (Celgard 2400). The cycling and rate capability tests were performed using a CT2001A battery program control test system within the voltage range of 0.01–1.00 V. The CV curves were tested in the voltage range of 0.01–3.00 V by a CHI 760E electrochemical workstation. Before tests, the coin‐type half‐cells were activated for 2 cycles at 100 µA between 0.01 and 1.00 V to exclude the potential adverse effect of contaminants or side reactions.

### Battery Simulation

The lithium‐ion battery interface of Comsol Multiphysics software was used for simulation, and the electrolyte node was used to define the battery electrolyte charge and ion transmission. Two electrode nodes were used to define the ohmic potential drop due to current conduction in each electrode phase. On the internal boundary between the electrode and electrolyte phase, the internal electrode surface nodes were used to define the charge transfer reaction. The transfer of solid lithium in the electrode phase was simulated by a single dilute substance transfer interface, in which the molecular flux of lithium was defined according to Fick's law. The electrode surface coupling node defined the molecular flux caused by the electrochemical reaction on the outer boundary of electrode particles. The concentration of solid lithium was dynamically coupled with the insertion of lithium ions into the reaction electrode, which was defined in the electrode reaction sub‐node of the internal electrode surface node. With the increase of lithium concentration in the Si/C anode material, volume expansion occurred. This phenomenon was simulated by Solid Mechanics interface, in which expansion was defined as a prestrain in the prestress and prestrain nodes (sub‐nodes of the linear elastic material node). Based on experimental data, the expansion was defined as a function of local solid lithium concentration by an interpolation method.

## Conflict of Interest

The authors declare no conflict of interest.

## Supporting information

Supporting Information

## Data Availability

Research data are not shared.

## References

[1] M. Park , X. Zhang , M. Chung , G. B. Less , A. M. Sastry , J. Power Sources 2010, 195, 7904.

[2] A. Van der Ven , J. Bhattacharya , A. A. Belak , Acc. Chem. Res. 2013, 46, 1216. 22584006 10.1021/ar200329r

[3] J. Ma , C. Wang , S. Wroblewski , J. Power Sources 2007, 164, 849.

[4] M. Weiss , R. Ruess , J. Kasnatscheew , Y. Levartovsky , N. R. Levy , P. Minnmann , L. Stolz , T. Waldmann , M. Wohlfahrt‐Mehrens , D. Aurbach , M. Winter , Y. Ein‐Eli , J. Janek , Adv. Energy Mater. 2021, 11, 2101126.

[5] Y. Tang , Y. Zhang , W. Li , B. Ma , X. Chen , Chem. Soc. Rev. 2015, 44, 5926. 25857819 10.1039/c4cs00442f

[6] Y. Wu , X. Huang , L. Huang , J. Chen , Energy Environ. Mater. 2021, 4, 19.

[7] S. Wen , B. Liu , W. Li , T. Liang , X. Li , D. Yi , B. Luo , L. Zhi , D. Liu , B. Wang , Adv. Funct. Mater. 2022, 32, 2203960.

[8] M. T. McDowell , S. W. Lee , W. D. Nix , Y. Cui , Adv. Mater. 2013, 25, 4966. 24038172 10.1002/adma.201301795

[9] Y. Wang , Z. Wang , F. Zheng , J. Sun , J. A. S. Oh , T. Wu , G. Chen , Q. Huang , M. Kotobuki , K. Zeng , L. Lu , Adv. Sci. 2022, 9, 2105849. 10.1002/advs.202105849 PMC9069353 35253384

[10] R. Li , G. Zhang , Y. Wang , Z. Lin , C. He , Y. Li , X. Ren , P. Zhang , H. Mi , Nano Energy 2021, 90, 106591.

[11] W. Li , S. Zhang , W. Zheng , J. Ma , L. Li , Y. Zheng , D. Sun , Z. Wen , Z. Liu , Y. Wang , G. Zhang , G. Cui , Adv. Funct. Mater. 2023, 33, 2300791.

[12] R. Li , G. Zhang , P. Zhang , Y. Li , C. He , X. Ren , H. Mi , Chem. Eng. J. 2022, 450, 138019.

[13] L. Bai , Z. Hu , C. Hu , S. Zhang , Y. Ying , Y. Zhang , L. Li , H. Zhang , N. Li , S. Shi , S. Liu , L. Hao , T. Liu , H. Huang , H. Huang , Y. Zhang , Angew. Chem., Int. Ed. 2023, 62, e202301631. 10.1002/anie.202301631 37017994

[14] P. K. Panda , J. Mater. Sci. 2009, 44, 5049.

[15] A. Koka , H. A. Sodano , Nat. Commun. 2013, 4, 2682. 24177706 10.1038/ncomms3682

[16] B.‐S. Lee , J. Yoon , C. Jung , D. Y. Kim , S.‐Y. Jeon , K.‐H. Kim , J.‐H. Park , H. Park , K. H. Lee , Y.‐S. Kang , J.‐H. Park , H. Jung , W.‐R. Yu , S.‐G. Doo , ACS Nano 2016, 10, 2617. 26815662 10.1021/acsnano.5b07674

[17] H. Mi , Y. Wang , H. Chen , L. Sun , X. Ren , Y. Li , P. Zhang , Nano Energy 2019, 66, 104136.

[18] S. Xia , Y. Zhao , J. Yan , J. Yu , B. Ding , ACS Nano 2021, 15, 3161. 33496181 10.1021/acsnano.0c09745

[19] K. Wu , J. Yi , X. Liu , Y. Sun , J. Cui , Y. Xie , Y. Liu , Y. Xia , J. Zhang , Nano‐Micro Lett. 2021, 13, 79. 10.1007/s40820-021-00599-2 PMC8187518 34138325

[20] P. Zou , R. Zhang , L. Yao , J. Qin , K. Kisslinger , H. Zhuang , H. L. Xin , Adv. Energy Mater. 2021, 11, 2100982.

[21] Y. Guo , R. Wang , C. Cui , R. Xiong , Y. Wei , T. Zhai , H. Li , Nano Lett. 2020, 20, 7680. 32881528 10.1021/acs.nanolett.0c03206

[22] Y. Zhang , X. Li , E. Sivonxay , J. Wen , K. A. Persson , J. T. Vaughey , B. Key , F. Dogan , Adv. Energy Mater. 2021, 11, 2101820.

[23] J. Lin , H. Peng , J.‐H. Kim , B. R. Wygant , M. L. Meyerson , R. Rodriguez , Y. Liu , K. Kawashima , D. Gu , D.‐L. Peng , H. Guo , A. Heller , C. B. Mullins , ACS Appl. Mater. Interfaces 2020, 12, 18465. 32223176 10.1021/acsami.9b23106

[24] C. Jiang , L. Xiang , S. Miao , L. Shi , D. Xie , J. Yan , Z. Zheng , X. Zhang , Y. Tang , Adv. Mater. 2020, 32, 1908470. 10.1002/adma.201908470 32108386

[25] Y. Huang , H. Yang , T. Xiong , D. Adekoya , W. Qiu , Z. Wang , S. Zhang , M. S. Balogun , Energy Storage Mater. 2020, 25, 41.

[26] S. Zhou , P. Huang , T. Xiong , F. Yang , H. Yang , Y. Huang , D. Li , J. Deng , M. S. Balogun , Small 2021, 17, 2100778. 10.1002/smll.202100778 34060232

[27] G. G. Eshetu , H. Zhang , X. Judez , H. Adenusi , M. Armand , S. Passerini , E. Figgemeier , Nat. Commun. 2021, 12, 5459. 34526508 10.1038/s41467-021-25334-8 PMC8443554

[28] L. Sun , Y. Liu , R. Shao , J. Wu , R. Jiang , Z. Jin , Energy Storage Mater. 2022, 46, 482.

[29] C. Lv , Z. Tong , Z.‐P. Wu , F. Gao , S.‐Y. Zhou , P.‐F. Zhang , Z.‐H. Zhou , H. G. Liao , Y. Zhou , S.‐G. Sun , J. T. Li , Energy Storage Mater. 2022, 51, 361.

[30] J. Xu , Q. Yin , X. Li , X. Tan , Q. Liu , X. Lu , B. Cao , X. Yuan , Y. Li , L. Shen , Y. Lu , Nano Lett. 2022, 22, 3054. 35315677 10.1021/acs.nanolett.2c00341

[31] W. Qian , W. Yang , Y. Zhang , C. R. Bowen , Y. Yang , Nano‐Micro Lett. 2020, 12, 149. 10.1007/s40820-020-00489-z PMC7770897 34138166

[32] M.‐S. Balogun , H. Yang , Y. Luo , W. Qiu , Y. Huang , Z.‐Q. Liu , Y. Tong , Energy Environ. Sci. 2018, 11, 1859.

[33] M. Guo , G.‐H. Kim , R. E. White , J. Power Sources 2013, 240, 80.

[34] P. Albertus , J. Couts , V. Srinivasan , J. Newman , J. Power Sources 2008, 183, 771.

[35] J. B. Siegel , A. G. Stefanopoulou , P. Hagans , Y. Ding , D. Gorsich , J. Electrochem. Soc. 2013, 160, A1031.

[36] V. Malave , J. R. Berger , P. A. Martin , J. Appl. Mech. 2014, 81, 091005.

[37] Y.‐T. Cheng , M. W. Verbrugge , J. Power Sources 2009, 190, 453.

[38] S. Pan , J. Han , Y. Wang , Z. Li , F. Chen , Y. Guo , Z. Han , K. Xiao , Z. Yu , M. Yu , S. Wu , D.‐W. Wang , Q.‐H. Yang , Adv. Mater. 2022, 34, 2203617. 10.1002/adma.202203617 35679574

[39] R. Zhu , L. Li , Z. Wang , S. Zhang , J. Dang , X. Liu , H. Wang , ACS Nano 2022, 16, 1119. 34936340 10.1021/acsnano.1c08866

[40] Y. Wakayama , T. Inokuma , S. Hasegawa , J. Cryst. Growth 1998, 183, 124.

[41] H. Zhao , K. Liang , S. Wang , Z. Ding , X. Huang , W. Chen , Y. Ren , J. Li , Adv. Sci. 2023, 10, 2303696. 10.1002/advs.202303696 PMC10582439 37607121

[42] H. Huang , X. Wei , S. Gao , Electrochim. Acta 2016, 220, 427.

[43] X. Zhang , D. Wang , X. Qiu , Y. Ma , D. Kong , K. Müllen , X. Li , L. Zhi , Nat. Commun. 2020, 11, 3826. 32737306 10.1038/s41467-020-17686-4 PMC7395733

[44] S. B. Tang , M. O. Lai , L. Lu , J. Alloys Compd. 2008, 449, 300.

[45] V. Augustyn , J. Come , M. A. Lowe , J. W. Kim , P.‐L. Taberna , S. H. Tolbert , H. D. Abruna , P. Simon , B. Dunn , Nat. Mater. 2013, 12, 518. 23584143 10.1038/nmat3601

[46] Y. Gao , B. Wang , Prog. Nat. Sci.: Mater. Int. 2023, 33, 203.

[47] T. Brezesinski , J. Wang , S. H. Tolbert , B. Dunn , Nat. Mater. 2010, 9, 146. 20062048 10.1038/nmat2612

[48] Y. Zhang , M. Xie , V. Adamaki , H. Khanbareh , C. R. Bowen , Chem. Soc. Rev. 2017, 46, 7757. 29125613 10.1039/c7cs00387k

[49] J. Frantti , J. Phys. Chem. B 2008, 112, 6521. 18433161 10.1021/jp711829t

[50] J. Chen , H. Zhao , J. Li , Y. Qi , K. Liang , L. Zhou , X. Huang , Y. Ren , Ceram. Int. 2022, 48, 11257.

