# Supplementary material for: Directional Polarization of a Ferroelectric Intermediate Layer Inspires a Built‐In Field in Si Anodes to Regulate Li+ Transport Behaviors in Particles and Electrolyte
Source: Adv Sci (Weinh). 2024 Apr 19;11(25):2402915. doi: 10.1002/advs.202402915 (PMC11220674; doi:10.1002/advs.202402915)
Supplement: Supplementary file 1 — Supporting Information [file ADVS-11-2402915-s001.docx]

***Supporting Information***

**Directional Polarization of a Ferroelectric Intermediate Layer Inspires a Built-in Field in Si Anodes to Regulate Li^+^ Transport Behaviors in Particles and Electrolyte**

M. Liu, W. Xu, S. Liu, B. Liu, Y. Gao, B. Wang

CAS Key Laboratory of Nanosystem and Hierarchical Fabrication

National Center for Nanoscience and Technology

Beijing 100190, P.R. China

E-mail: gaoyang@nanoctr.cn; wangb@nanoctr.cn

M. Liu, B. Liu, Y. Gao, B. Wang

University of Chinese Academy of Sciences

Beijing 100039, P. R. China

W. Xu

State Key Laboratory for Advanced Metals and Materials

School of Materials Science and Engineering

University of Science and Technology Beijing

Beijing 100083, P. R. China

S. Liu

Key Laboratory of Bio-based Material Science and Technology of Ministry of Education

Engineering Research Center of Advanced Wooden Materials of Ministry of Education

College of Material Science and Engineering

Northeast Forestry University

Harbin 150040, China


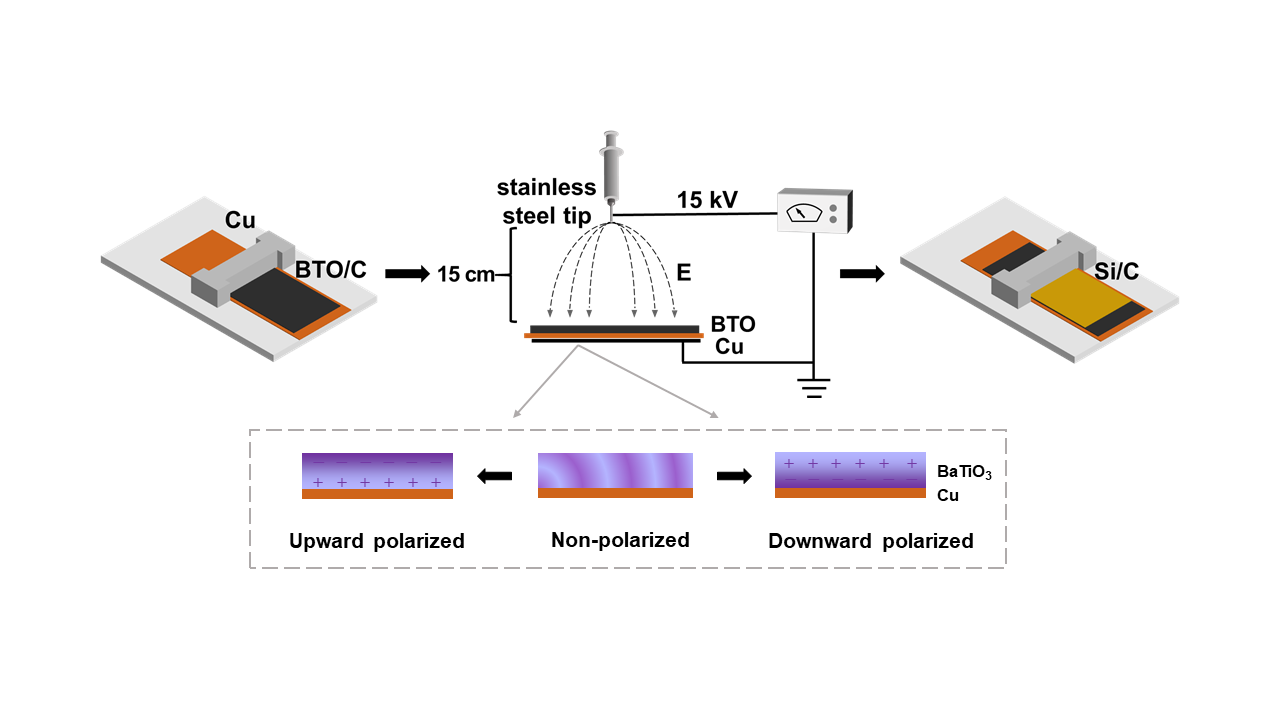


**Figure S1.** Schematic of the preparation process of double-layer electrodes, composed of a BaTiO_3_/C interlayer and a Si/C active layer, with different polarization directions.


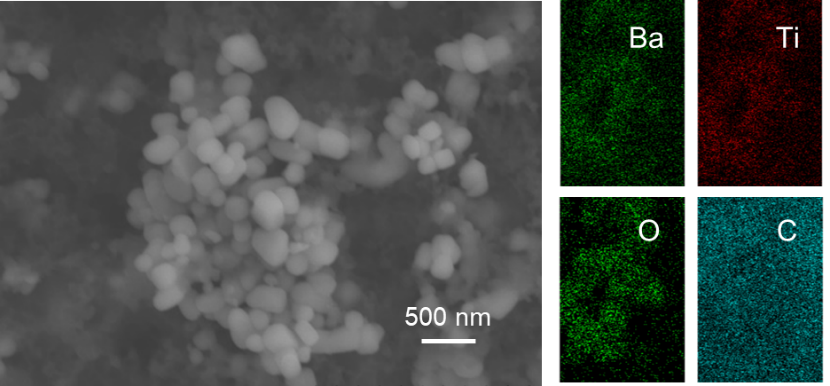


**Figure S2.** SEM images and corresponding elemental mapping of the BaTiO_3_/C.


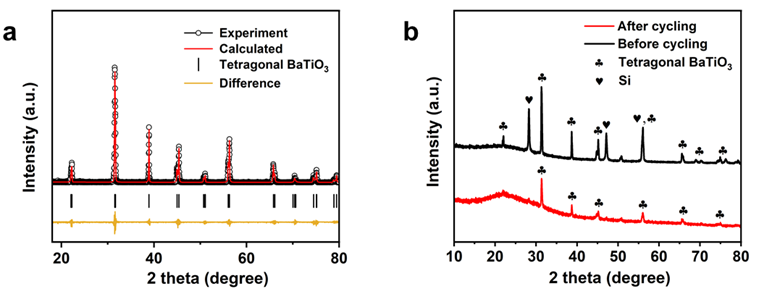


**Figure S3.** (a) XRD pattern of BaTiO_3_ powders. (b) XRD patterns of the double-layer electrodes before and after cycling.


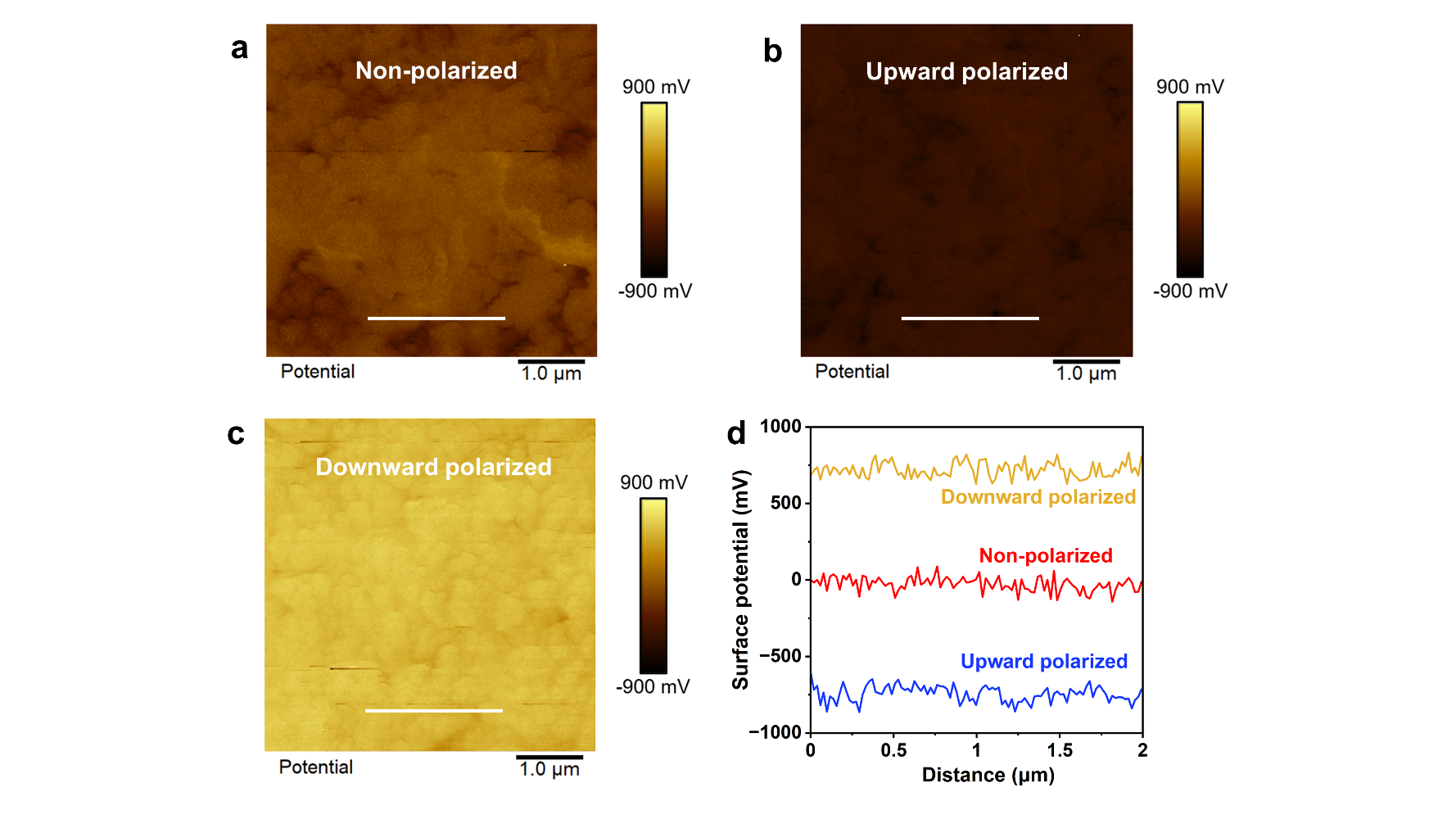


**Figure S4.** KPFM images of (a) non-polarized, (b) upward polarized, and (c) downward polarized BaTiO_3_/C layers over an area of 25 μm^2^. (d) The corresponding KPFM surface potential profiles of different polarized samples retrieved from the white line of each image.


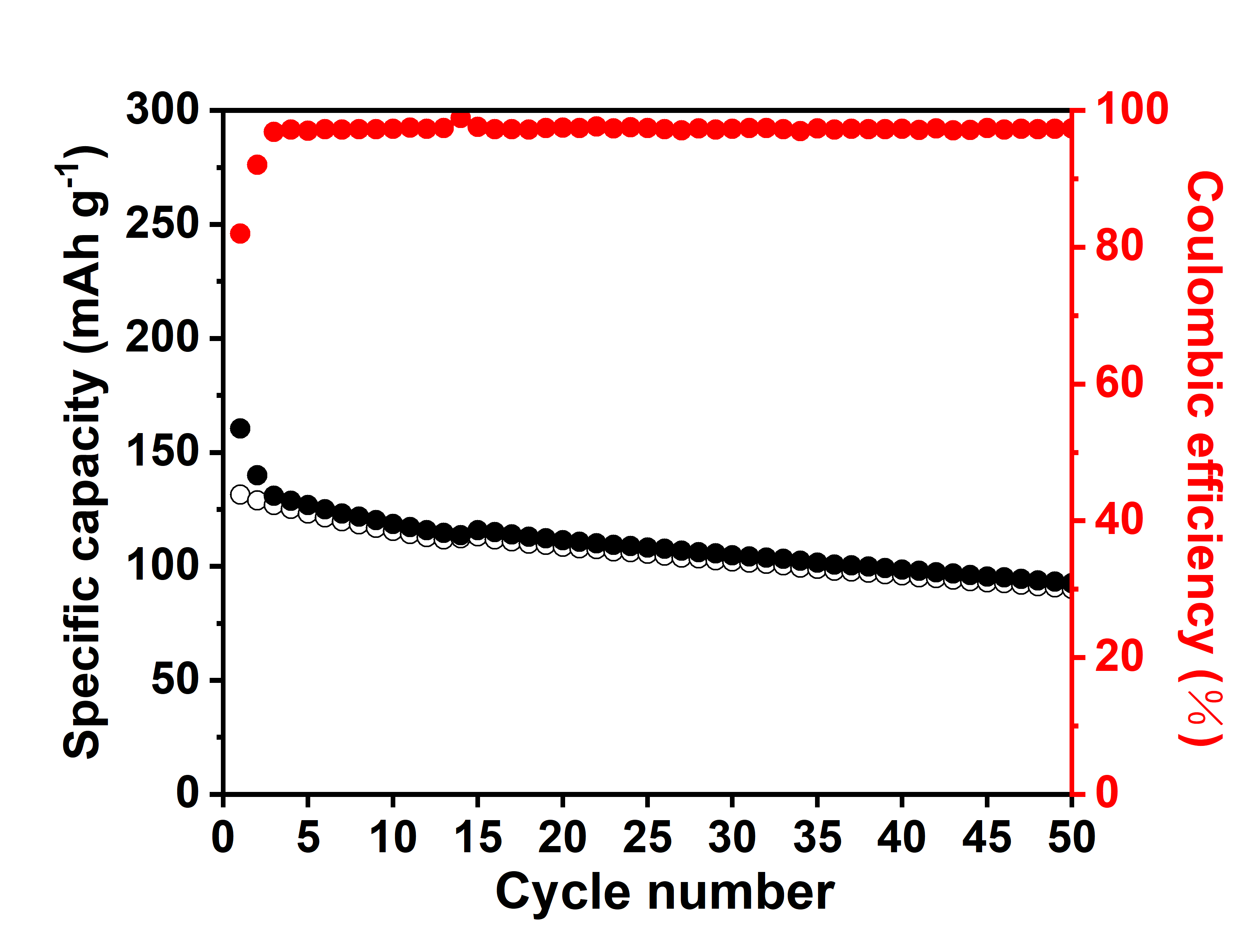


**Figure S5.** Cycling performance of LFP//up-polarized BaTiO_3_ full cell. Note that lithium iron phosphate (LFP) cathodes were fabricated by mixing commercial LFP, carbon black, and polyvinylidene fluoride (PVDF) in N-methyl-2-pyrrolidone (NMP) at a mass ratio of 8:1:1. The N/P ratio is ~1.1.


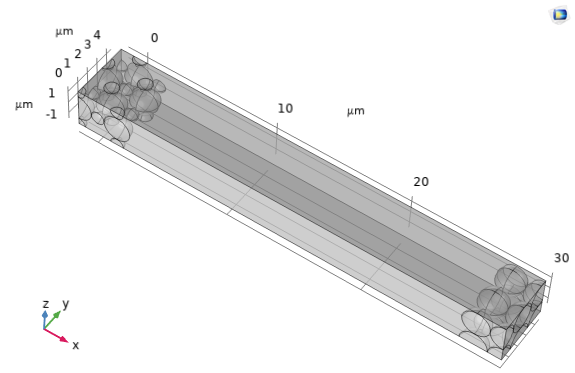


**Figure S6.** 3D simulation geometric model of a lithium-ion battery.


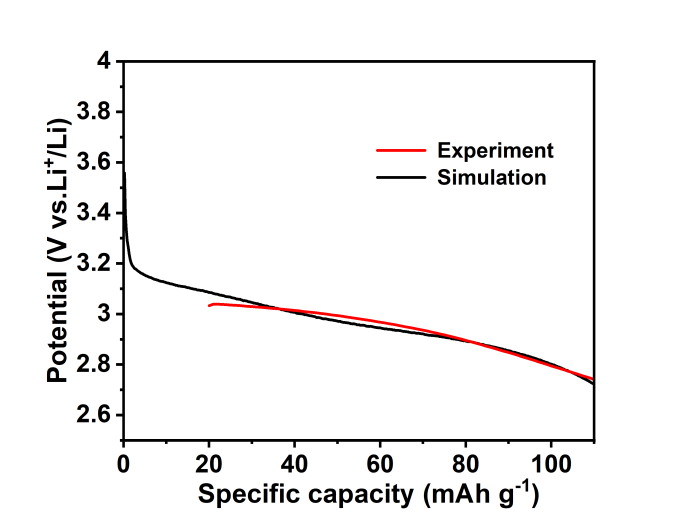


**Figure S7.** Experimental and simulated voltage profiles of LFP// up-polarized BaTiO_3_ full cell.


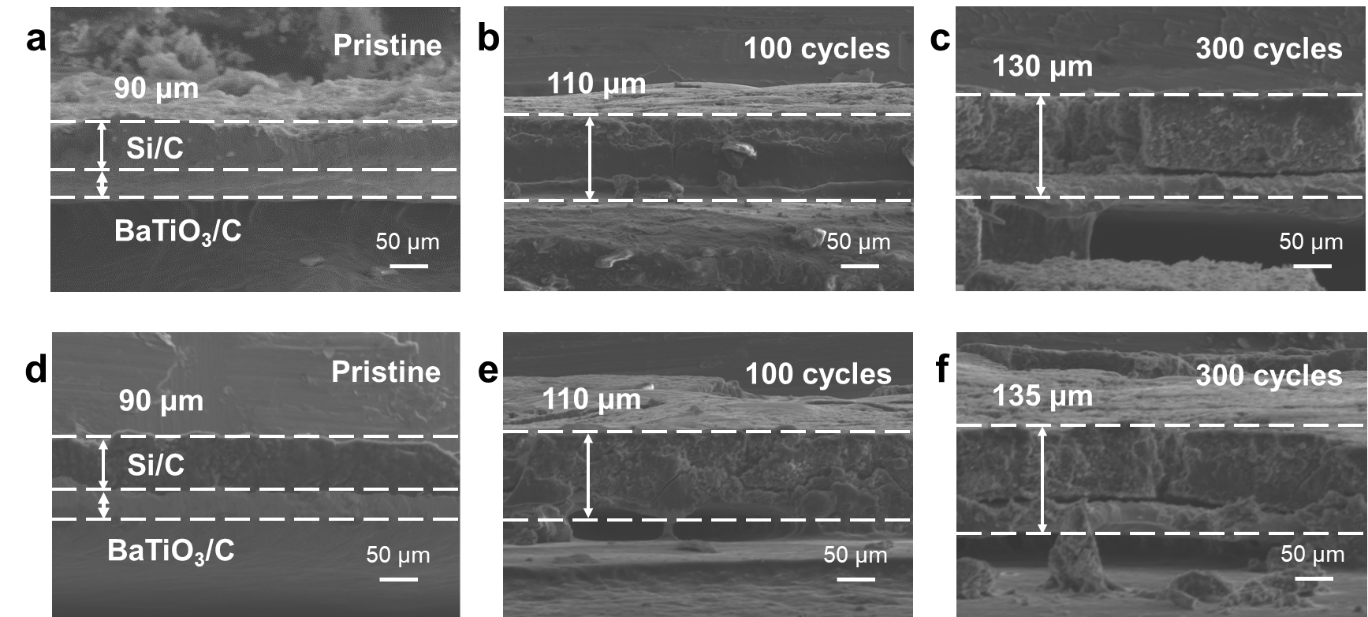


**Figure S8.** (a-c) SEM images of the upward polarized electrodes before and after different cycles at 2.1A g^−1^. (d-f) SEM images of the non-polarized electrodes before and after different cycles at 2.1A g^−1^.


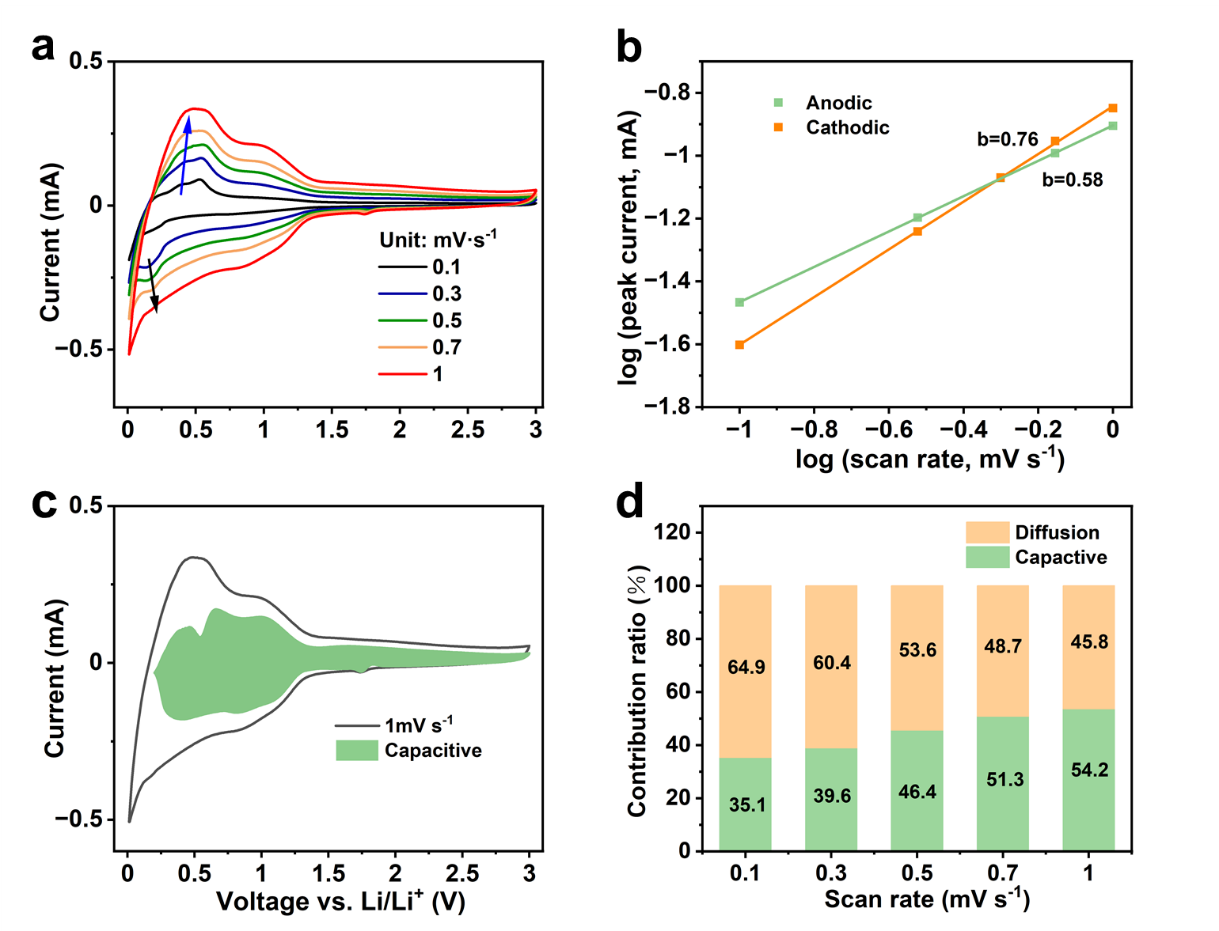


**Figure S9.** (a) CV profiles of downward polarized sample at different scan rates. (b) Correlations of peak current and scan rate. (c) CV curve and capacitive proportion to the charge storage of downward polarized sample at the scan rate of 1.0 mV s^−1^. (d) Normalized proportion ratio of capacitive and diffusion-controlled capacities at various scan rates.


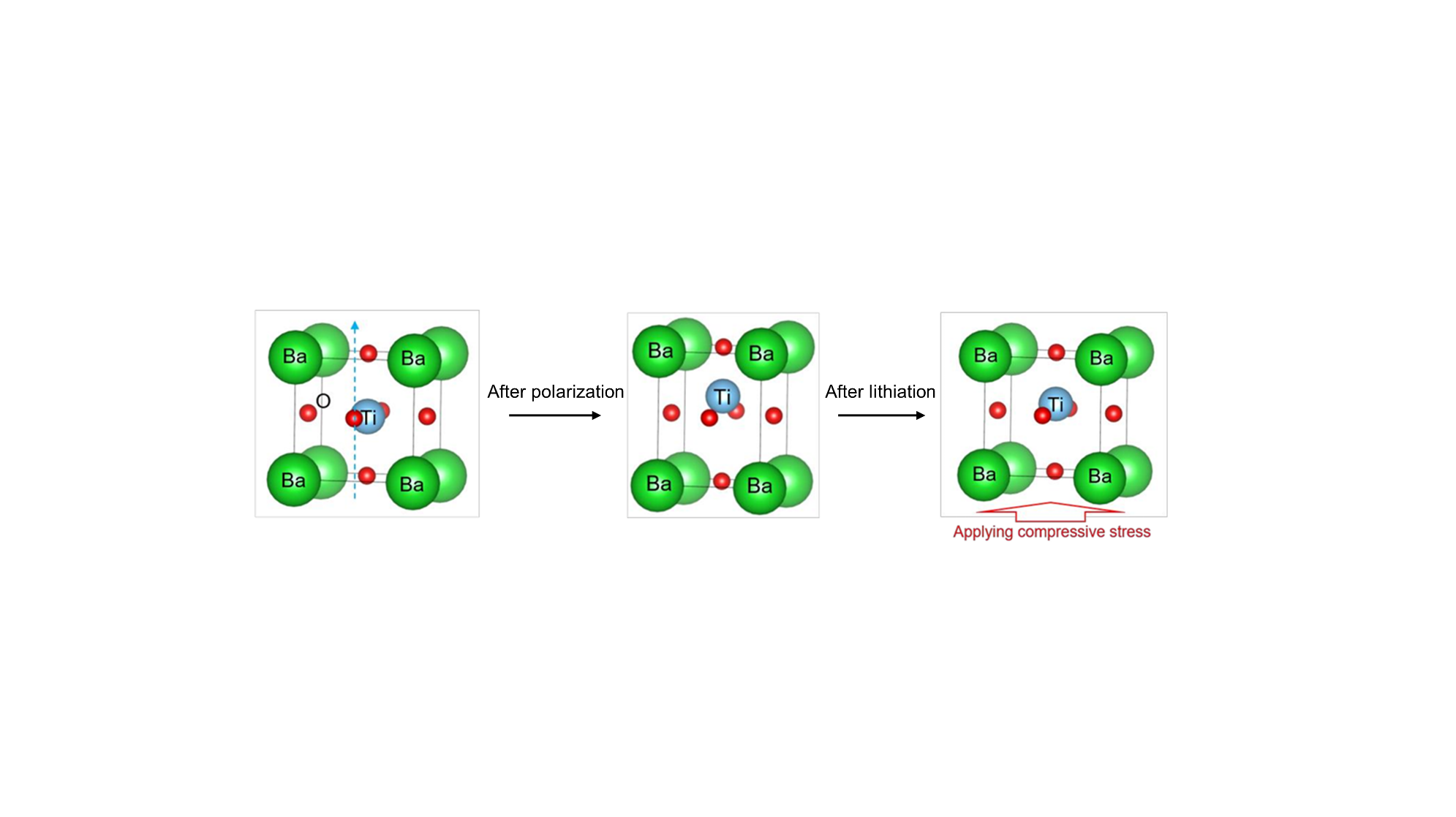


**Figure S10**. Schematic of Ti ion migration in the [TiO_6_] octahedral interstitial sites during polarization and lithiation (under the external compressive stress).

**
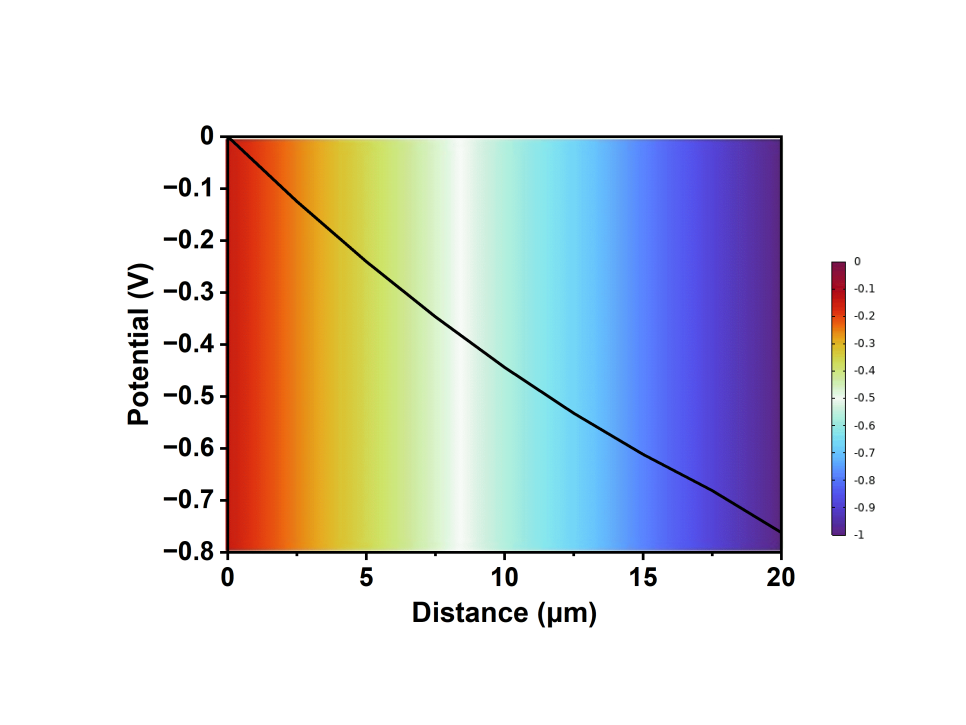
**

**Figure S11.** Potential change in the upward polarized BaTiO_3_ interlayer. Note that Cu foil is on the left.
